# *Helicobacter pylori* (*H. pylori*) Infection-Associated Anemia in the Asir Region, Saudi Arabia

**DOI:** 10.3390/diagnostics13142404

**Published:** 2023-07-18

**Authors:** Omar A. Al Mutawa, Mohammad Asrar Izhari, Raed A. Alharbi, Abdulmajeed Abdulghani A. Sindi, Abdullah M. Alqarni, Foton E. Alotaibi, Ahmed R. A. Gosady, Daifallah M. M. Dardari, Abdulrahman M. Almutairi, Mohammed Alshehri, Ahmed I. E. Athathi

**Affiliations:** 1 Medical Laboratory Department Southern Region Armed Forces Hospital, Khamis Mushait 62413, Saudi Arabia; 2 Department of Laboratory Medicine, Faculty of Applied Medical Sciences, Al-Baha University, Al-Baha 65528, Saudi Arabia; 3 Department of Basic Medical Sciences, Faculty of Applied Medical Sciences, Al-Baha University, Al-Baha 65528, Saudi Arabia; 4 Department of Genetic Counseling, Al-Faisal University, Riyadh 11533, Saudi Arabia; 5 Laboratory Department, Baish Primary Healthcare, Jazan 87386, Saudi Arabia; 6 Laboratory Department, Baish General Hospital, Jazan 87597, Saudi Arabia; 7 Health Facilities Infection Control Department, General Directorate of Health Al-Baha, Al-Baha 65522, Saudi Arabia; 8 Department of Community Health Sciences (Public Health), Faculty of Applied Medical Sciences, Al-Baha University, Al-Baha 65528, Saudi Arabia; 9 Laboratory Department, King Fahad Central Hospital, Jazan 85534, Saudi Arabia

**Keywords:** *Helicobacter pylori*, infection, IDA, anemia, logistic regression

## Abstract

*H. pylori* (ubiquitous) and anemia together represent one of the growing health concerns globally. Gastroduodenal sequelae of *H. pylori* infection are distinguished; however, for the *H. pylori* infection and its implication in the development of anemia, iron has a significant health impact. We aimed to evaluate *H. pylori* infection-associated anemia by employing a logistic regression analysis model. A retrospective (case–control) study design-based assessment of the *H. pylori* associated-anemia. The study area was geo-referenced by QGIS/QuickMapServies. Descriptive and inferential statistical analyses were accomplished using the R-base–R-studio (v-4.0.2)-tidyverse. A *p*-value < 0.05 was the statistical significance cut-off value. A ggplot2 package was used for data representation and visualization. Mean ± SD age, Hb, MCV, ferritin, and RBC for overall study participants were measured to be 44.0 ± 13.58, 13.84 ± 2.49, 83.02 ± 8.31, 59.42 ± 68.37, and 5.14 ± 0.75, respectively. Decreased levels of Hb (infected vs. uninfected: 13.26 ± 2.92 vs. 14.42 ± 1.75, *p* < 0.001) ferritin (infected vs. uninfected: 48.11 ± 63.75 vs. 71.17 ± 71.14, *p* < 0.001), and MCV (infected vs. uninfected: 81.29 ± 9.13 vs. and 84.82 ± 6.93, *p* < 0.05) were measured to be associated with *H. pylori* infection when compared with *H. pylori* uninfected control group. Moreover, the magnitude (prevalence) of anemia (infected vs. uninfected: 78% vs. 21%, *p* < 0.001), iron deficiency anemia (IDA) (infected vs. uninfected: 63.3% vs. 36.6%, *p* < 0.001), and microcytic anemia (infected vs. uninfected: 71.6% vs. 46.1%, *p* < 0.001) were significantly different among the *H. pylori*-infected participants. The higher likelihood of developing anemia (AOR; 4.98, 95% CI; 3.089–8.308, *p* < 0.001), IDA (AOR; 3.061, 95% CI; 2.135–4.416, *p* < 0.001), and microcytic anemia (AOR; 3.289, 95% CI; 2.213–4.949, *p* < 0.001) by 398%, 206.1%, and 229%, respectively, was associated with *H. pylori*-infected. We recommend the regular monitoring of hematological parameters and eradication of *H. pylori* infection to minimize the extra-gastric health consequences of *H. pylori* infection.

## 1. Introduction

*H. pylori* is a Gram-negative ubiquitous pathogen that poses a consequential health threat. Through the fecal-oral transmission route, *H. pylori* gastrointestinal infection is contracted in childhood and triggers prolonged gastric inflammatory complications in approximately 2–10% of infected persons [1]. Approximately 50% (~4.4 billion) of the global population is remarkably impacted by the *H. pylori* infection [2,3]. Following the colonization of gastric mucosa, *H. pylori* predominantly trigger long-term and progressive gastric inflammatory diseases such as gastric atrophy, gastritis, peptic ulcer diseases [4], gastrointestinal metaplasia [1], antrum gastritis [5], mucosa-associated lymphoid tissue (MALT) lymphoma or gastric cancer [6]. Moreover, *H. pylori* infection has been found to be implicated in gastric carcinogenesis [7] and various other gastroduodenal pathologies [8]. In addition to that, the occurrence of gastrointestinal malignancies [9,10] is linked with the carcinogenic nature of *H. Pylori*, which is categorized as a Class 1 carcinogen by the International Agency for Research on Cancer (IARC) [11]. Though high morbidity and mortality have been associated with *H. pylori* infection [8,12], however, almost 80% of infected individuals remain asymptomatic [13]. In addition to the gastroduodenal pathological implication of *H. pylori*, various types of extra-gastric manifestations: IDA [14], hypercholesterolemia [15], immune thrombocytopenia [16], lipid profile alteration [17], atherosclerosis-dependent cardiovascular disease (CVD), cerebral infarction and neurological disorders [2] have been reported in the literature. Furthermore, *H. pylori*-associated dermatological and ophthalmic diseases have also surfaced [2]. Evaluation of extra-gastric clinical impacts of *H. pylori* infection has been of paramount importance in dealing with the *H. pylori* infection and its extra-gastric effect on individuals effectively in recent years. Anemia, especially iron-deficiency anemia, is one of the most crucial *H. pylori* infection-associated extra-gastric diseases. However, *H. pylori* has also been found to be implicated in the development of other hematological disorders, such as idiopathic thrombocytopenic purpura (ITP) and pan gastritis [18].

Anemia is an important public health concern and affects a sizeable portion of the global population, as per WHO report [19]. The high worldwide prevalence of anemia (>30%) has impacted all age categories in both developed and developing countries [20]. The economic burden of anemia on the healthcare sector varies based on severity, type, and comorbidities [21]. Iron deficiency anemia (IDA) has been reported by Mubaraki et al. to be highly prevalent, especially in people of developing countries [19]. IDA has been recognized as the most frequent nutritional deficiency by the WHO, which affects a huge section (30%) of the global population [22]. Though IDA affects approximately 2–5 percent of the adult population in the developed world, however, 15% of unexplained IDA cases have also been delineated [23,24]. Owaidah et al. reported the impact of IDA on the cognitive, physiological, and physical capabilities of IDA patients [25]. Furthermore, the significant impact of IDA on quality of life has also been reported by Andro et al. [26]. Following the eradication of *H. pylori* infection, improvement in deteriorating hematological parameters such as ferritin level, hemoglobin, and iron of the infected patients, pointed out the association of *H. pylori* infection with the development of IDA for the first time in 1991, which highlights the relevance of the present study [27]. *H. pylori* colonizes mucosa and causes multiple micro-erosions resulting in chronic gastrointestinal bleeding, which could constitute one of the possible mechanisms of the development of *H. pylori*-associated IDA. Gastrointestinal bleeding [27], enhanced iron requirement, especially during pregnancy, hemorrhagic gastritis, reduced iron absorption, actively bleeding ulcers, reduced intake of dietary iron, and decreased iron absorption as in chronic intestinal infections and coeliac diseases have been the major etiology of IDA [22,28]. In addition, reduced gastrointestinal absorption triggered by edema and inflammatory responses is one of the major factors of IDA [29]. Low intra-gastric pH (acidic) fosters dietary iron reduction (Fe^3+^ to Fe^2+^), which is necessary for effective absorption [30]. *H. pylori* infection (chronic gastritis) causes gastric hypochlorohydria, which impairs dietary iron reduction (Fe^3+^ to Fe^2+^) and iron absorption leading to the development of IDA [31]. Furthermore, the sequestration of iron by *H. pylori* for its growth and enhanced release of acute phase reactant (hepcidin) in response to gastric mucosal inflammatory process triggers the IDA of chronic diseases [32]. Additionally, certain membrane-bound proteins of *H. pylori* such as cytotoxin-associated gene A (CagA), heat shock proteins (HSPS), and vacuolization cytotoxin A (VacA), plays a key role in the pathogenesis and development of IDA in the patients [33,34]. Development of *H. pylori* infection associated with IDA is noteworthy as both *H. pylori* infection and IDA are the most common health problems. An inquisitive assessment of the association of *H. pylori* infection-associated anemia, particularly IDA, is worthy and justified to be addressed to foster effective management of IDA in *H. pylori*-infected patients. Therefore, this study aimed to evaluate the impact of *H. pylori* infections on the development of IDA in the Asir population, Kingdom of Saudi Arabia.

## 2. Materials and Methods

### 2.1. Ethical Approval and Declaration

We followed the guidelines of the Declaration of Helsinki to accomplish this study. Ethical approval was issued to this study by the Research Ethical Committee (REC), Armed Forces Hospital Southern region, Kingdom of Saudi Arabia on 2 April 2023 (Reference number: AFHSRMREC/2023/687). Data confidentiality and the purpose of the study were described to the Committee in written consent before the collection of data included in the study and commencement of the study. Data were reviewed and extracted from medical records at Armed Forces Hospital Southern Region, KSA, as per the policy laid down by REC.

### 2.2. Study Area, Study Design, and Population

**Figure 1:**
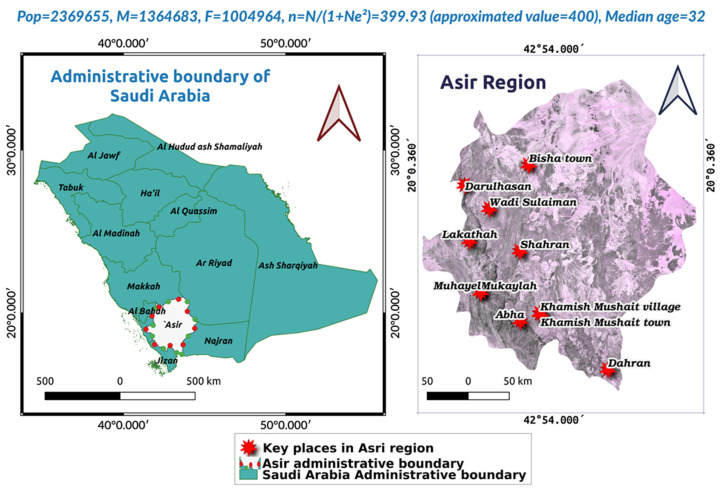
Comprehensive study area map illustrating the study location, population size, and approximated sample size needed for the study. N: population size; n: sample size; e: margin of error (0.05); Pop: overall population; M: male population; F: female population in Asir geographical region of Saudi Arabia.

**Figure 2:**
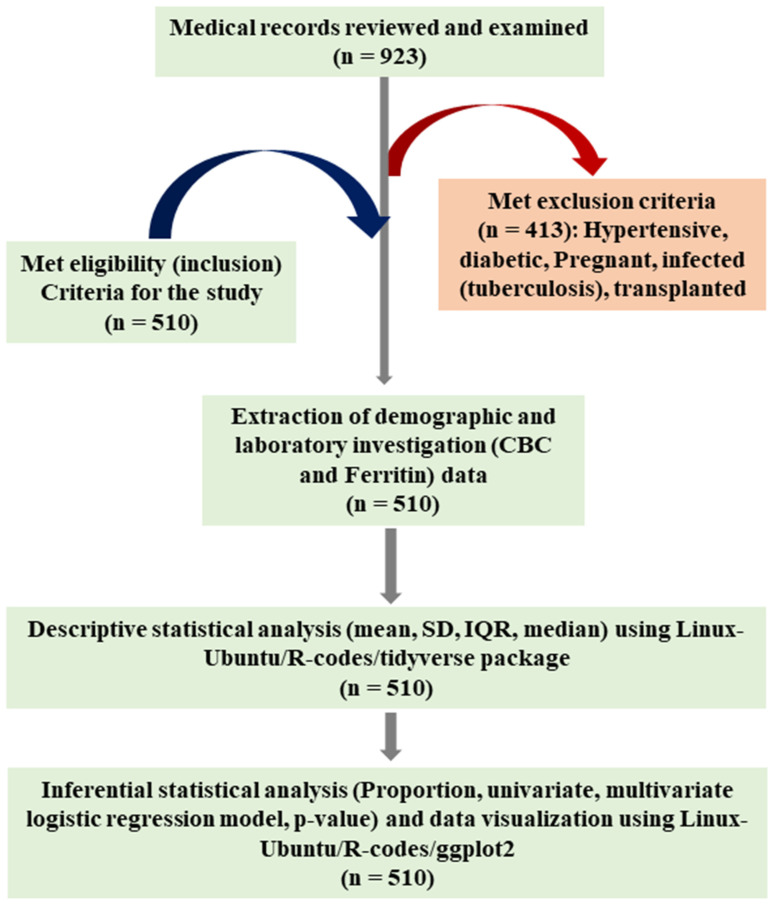
Illustration of summary of the materials and methods used in this study: sample size, hematological study parameters, statistical models, and data visualization tools.

A case–control (Hospital-based observational) study was conceptualized to carry out this study. *H. pylori*-infected and non-infected participants were considered as case and control, respectively, to determine the *H. pylori* infection associated with IDA in the Asir population [35]. Asir region of KSA is located in the southwest, which has been well illustrated by developing an all-inclusive study area map (Figure 1). We used QGIS (V3.30.2) and QuickMapServies accessory plug-ins to obtain insight into geo-reference [36]. We inquisitively examined the laboratory test reports of *H. pylori*-infected as well as *H. pylori* uninfected study participants (both outpatients and inpatients), which were available at Armed Forces Hospitals of Southern (Asir) part of KSA from 1 June 2017 to 27 March 2023. Most of the study subjects included in the current study were Saudi nationals. The laboratory investigation reports were scrutinized, reviewed, and screened for demographic details (age and gender), complete blood count (CBC), and level of ferritin [35]. Relevant data that met the inclusion criteria were plugged into the Excel sheet (MS Office 365) to clean, stratify, and process. Medical laboratory reports from King Faisal Military Hospital, Military; Family and Community Medical Center Asir, Base Khamis Mushait; King Fahad Military Hospital—Khamis Mushait, Khamis Mushait City; Family and Community Medical Center—Abha, Abha City; and Family and Community Medical Center—Ahad Rafidah, Ahad, Rafida province, were included in present investigation for data analyses. Various steps of data processing methods have been elucidated in Figure 2.

### 2.3. Eligibility/Inclusion Criteria

*H. pylori*-infected and *H. pylori*-uninfected study participants of both the gender and all age groups were encompassed in the present study.

### 2.4. Eligibility/Exclusion Criteria

Hospital records of participants who contracted tuberculosis, diabetes, and hypertension were scrutinized and excluded from the study. Furthermore, participants on antiviral treatment, women with pregnancy, kidney transplants, and other multiple comorbidities were inquisitively examined and excluded.

### 2.5. Assessment of Sample Size

For the commencement of this study, we calculated the sample size using n = N/(1 + Ne^2^) statistical equation where n = sample size, N = population, e = margin of error considering 95% Confidence level (95% CI), and margin of error 0.05 [37]. Almost the sample size (n = 400) was assessed to be appropriate for the present study (Figure 1). Based on the sample size assessed, we studied the n = 510 participants to compensate for the sampling variability (Figure 2).

### 2.6. Operational Description

World Health Organization (WHO) defines anemia as per hemoglobin (Hb) concentration < 12.00 g/dL in non-pregnant women and <13.00 g/dL in men [23]. Ferritin concentration < 30 μg/L was used for the determination of IDA [38]. MCV is a well-characterized a priori as microcytic anemia with MCV value < 80 fL, normocytic anemia with MCV = 80–100 fL, and macrocytic anemia with MCV > 100 fL [39].

### 2.7. Data Generation Strategies and Laboratory Techniques Applied

We reviewed, scrutinized, and encompassed the demographic data of all participants from a sum of n = 923 medical laboratory records of hospitals, which were taken under consideration for carrying out this study. The number of excluded participants was n = 413, which did not meet the inclusion criteria (Figure 1). Principally, blood and fecal specimens were collected at respective sample collection centers of the respective hospitals encompassed in this study as per the standard operating procedures (SOPs) of the hospitals. Blood and fecal specimens were further processed in medical laboratories. CBC (MCH-pg, MCHC-g/dL, MCV-fL, PLT-10^9^/L, WBC-10^9^/L and RBC-10^12^/L) was assessed by ADVIA 2120i hematological system. Ferritin was also assessed by using DxI 800 auto-analyzer device (Beckman Coulter, Brea, CA, USA). The rapid immune-chromatographic technique was employed to detect antigens of *H. pylori* in processed fecal specimens to confirm the *H. pylori*-infected individuals. For rapid immuno-chromatographic tests, Immuno CARD STAT and HPSA TEST PROCEDURE were employed. Stool samples were processed according to the manufacturer’s instructions to obtain the primary data on the diagnosis. The test is based on a combination of anti-human–Ig dye conjugate and purified *H. pylori* proteins. When the diluted stool specimen flows through absorbent immuno-chromatographic devices, the anti-human–Ig–IgG complex binds to the *H. pylori* proteins (Ags) fixed in the absorbent device to produce a colored band.

### 2.8. Quality Management of the Data

The data collector/investigator scrutinized the records inquisitively to remove technical bias, if any. Many reviews were made to ensure the quality and completeness of demographic and medical laboratory investigation data. Discrepant and/or incomplete cases were removed from this study.

### 2.9. Analyses and Interpretation of Stratified Data

We stratified the collected and quality-checked data of all study participants by gender (male and female) and different age categories. We stratified the data into three age groups: aged < 30 years, age = 30–60 years, and age > 60 years before data analyses were executed. *H. pylori* antigen-positive and -negative participants were treated as case and control, respectively. Descriptive data analysis was undertaken by applying R-base/R-studio (v-4.0.2). Continuous and categorical variables were analyzed by using Ubuntu/Linux-based machine. Descriptive statistical analyses of continuous variables included measurement of the mean, median, and interquartile range (IQR) of each parameter for all the participants in all stratified groups. While analyses of categorical variables (chi-square test and two-sample proportion test) were properly summarized in the form of frequency as well as proportion/percentage. We employed R-base/R-studio (v-4.0.2)/tidyverse package for executing inferential analyses (*t*-test, univariate and multivariate statistical logistic regression test for association). We considered *p*-values < 0.05 as the cut-off level for considering statistical significance. For plotting and data visualization, various packages and dependency packages of R were used.

## 3. Results

### 3.1. Baseline Features of the Hematological (CBC and Ferritin) Parameters of Study Participants (n = 510)

Retrieved continuous data were stratified into six distinct categories: overall, male, female, age < 30 years, age = 30–60 years, and age > 60 years before performing relevant statistical analyses. In totality, nine observations: age, Hb, MCV, ferritin, MCH, MCHC, RBC, WBC, and platelets, were included in deep data analyses. Mean ± SD age, Hb, MCV, ferritin, MCH, MCHC, RBC, WBC, and platelets for overall study participants were measured to be 44.0 ± 13.58, 13.84 ± 2.49, 83.02 ± 8.31, 59.42 ± 68.37, 26.8 ± 3.36, 31.75 ± 2.1, 5.14 ± 0.75, 5.99 ± 1.71, and 270.2 ± 74.23, respectively (Table 1). Moreover, median (IQR) age, Hb, MCV, ferritin, MCH, MCHC, RBC, WBC, and platelets for overall categories was observed to be 43 (53–35.0), 14.2 (15.6–12.3), 24.5 (89–12), 84.02 (89.1–78.1), 27.5 (29.1–25.5), 31.9 (32.9–30.9), 5.19 (5.66–4.68), 5.69 (6.9–4.74), and 262.0 (307–220), respectively (Table 1). Baseline characteristics of all the nine parameters by gender and various age categories are elaborated in Table 1.

### 3.2. Correlational Analyses of All the Hematological Parameters of Study Participants (n = 510) by H. pylori Infection Status (Case vs. Control)

**Figure 3:**
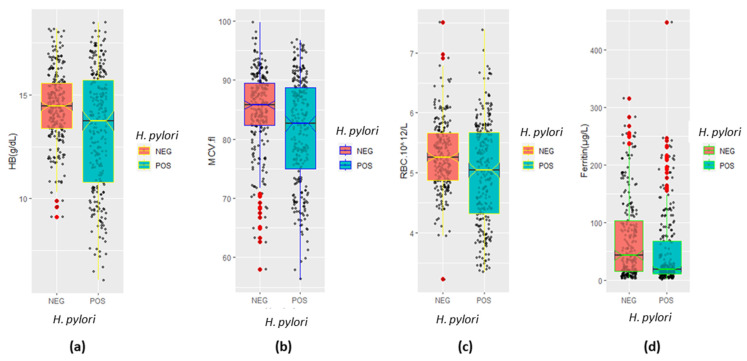
Portray of key hematological parameters by *H. pylori* infection status: (**a**) hemoglobin by *H. pylori* infection, (**b**) level of MCV in femtoliter by *H. pylori* infection status, (**c**) RBC disaggregated by *H. pylori* infection (**d**) Ferritin distribution by *H. pylori* infection.

**Figure 4:**
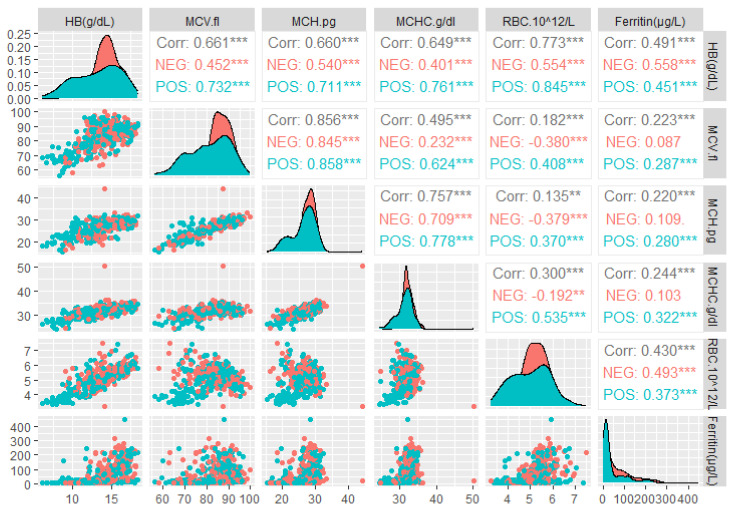
Correlelogram of significant hematological parameters by *H. pylori* infection status: correlation coefficients (corr) shown in a grey shade, red, and cyan color describes the correlation between hematological parameters in overall *H. pylori* control and *H. pylori* case study participants, respectively. Each asterisk (*) denotes the level of statistical significance.

The comparative status of vital hematological parameters of *H. pylori*-infected and *H. pylori*-uninfected study subjects was assessed, and the *p*-value was deduced for each parameter to comprehend the statistically significant difference. Comprehensive boxplot and correllelogaram were used to elucidate and illustrate the correlation between the parameters among *H. pylori* positive and negative study participants (Figure 3 and Figure 4). The mean ± SD of hemoglobin in the *H. pylori*-infected subject was 13.26 ± 2.92 (95% CI; 0.74–1.57), which was significantly (*p* < 0.001) lower than that in the *H. pylori* uninfected subject 14.42 ± 2.92 (95% CI; 0.74–1.57) as tabulated in Table 2. In addition, the overall mean ± SD of ferritin in *H. pylori*-infected vs. uninfected was 48.11 ± 63.75, 95% CI; 11.30–34.83 vs. 71.17 ± 71.14, 95% CI; 11.30–34.83, and the difference in the level of ferritin among the two groups were statistically significant (*p* < 0.001) (Table 2).

A statistically significant difference was also recorded in the level of MCV by *H. pylori* infection status (*p* < 0.001), and mean ± SD MCV in *H. pylori* case (infected) and *H. pylori* control (uninfected) was 81.29 ± 9.13, 95% CI; 2.12–4.93 and 84.82 ± 6.93, 95% CI; 2.12–4.93, respectively (Table 2). Our results of the *t*-test show the depression in the level of hemoglobin, ferritin, and MCV to the extent of statistical significance (*p* < 0.05) in *H. pylori*-infected individuals in comparison to control (*H. pylori* uninfected individuals) for overall category of the data. Mean ferritin level in case (*H. pylori* positive) versus control (*H. pylori* negative) in female and age > 60 years participant was 23.06 ± 34.3, 95% CI; 1.40–14.60 vs. 29.65 ± 30.0, 95% CI; 1.40–14.60 and 43.43 ± 56.50, 95% CI; 7.68–47.55 vs. 63.36 ± 53.12, 95% CI; 7.68–47.55, respectively (Table 2). Mean ferritin in females and age > 60 years was statistically insignificant (*p* > 0.05). The differences in the level of each hematological parameter of *H. pylori*-infected participants for the age > 60 years category were statistically insignificant (*p* > 0.05) as compared to the control (Table 2). The distribution of Hb, MCV, RBC, and ferritin data by *H. pylori* infection status (positive vs. negative) was found to be distinct, which is illustrated in Figure 3. We deduced a comprehensive correllelogaram to demonstrate the correlations between the hematological parameters of the study participants for overall *H. pylori*-infected and *H. pylori*-uninfected individuals (Figure 4). The correlation between Hb and ferritin for overall, *H. pylori*-positive, and *H. pylori*-negative was measured to be r = 0.491 (*p* < 0.001), r = 0.451 (*p* < 0.001), and r = 0.558 (*p* < 0.001), respectively, which shows the moderately positive correlation between the two parameters (Figure 4). A comparatively lesser positive correlation was observed between MCV and ferritin for overall (r = 0.22, *p* < 0.001) and *H. pylori*-positive (r = 0.287, *p* < 0.001), while a statistically insignificant correlation was noticed in *H. pylori*-negative (r = 0.087, *p* > 0.05) individuals. Similarly, the overall correlation between Hb and MCV has been recorded to be r = 0.66, *p* < 0.001, whereas that for *H. pylori*-positive and *H. pylori*-negative was evaluated to be r = 0.732, *p* < 0.001 and r = 0.452, *p* < 0.001 accordingly (Figure 4).

### 3.3. Magnitude of Anemia, IDA, and Anemia Morphotype in All the Study Participants (n = 510)

**Figure 5:**
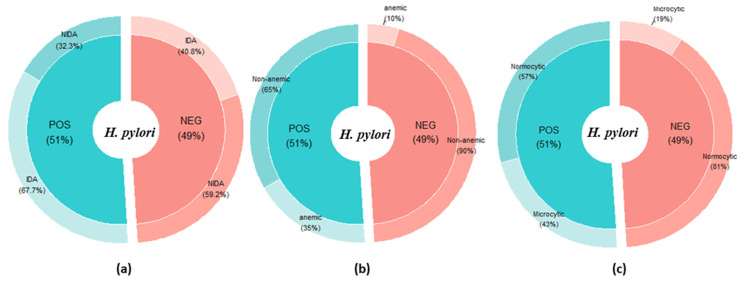
Prevalence of anemia by *H. pylori* infection status: (**a**) prevalence of iron deficiency anemia (IDA) by *H. pylori* infection; (**b**) prevalence of anemia (*H. pylori* positive versus *H. pylori* negative); (**c**) prevalence of anemia morphotypes by *H. pylori* infection.

The magnitude of anemia, iron deficiency anemia, and anemia morphotype in terms of proportion was evaluated by employing a two-sample test (for equality of proportions) and Pearson’s Chi-squared test (to assess *p*-value) for all the stratified groups of the participants (Table 3), and the proportion determined was based on conditional distribution. Moreover, the proportion of anemia, iron deficiency anemia, and morphotype was also assessed based on the marginal distribution (Figure 5). For the overall category, the significant (*p* < 0.001) difference in anemia prevalence (case vs. control) was 78% (N; 90, 95% CI; 0.27–0.44) vs. 21% (N; 24, 95% CI; 0.27–0.44) (Table 3). The overall prevalence of IDA in case and control was observed to be 63.3% (N; 176, 95% CI; 0.18–0.35) and 36.6% (N; 102, 95% CI; 0.18–0.35), and the difference was statistically significant (*p* < 0.001). Moreover, the overall prevalence of anemia was assessed to be 78% (N; 90, 95% CI; 0.27–0.44) and 21% (N; 24, 95% CI; 0.27–0.44), and the difference in proportion between the two groups was statistically significant (*p* < 0.001). A significant difference (*p* < 0.001) in the overall proportions of microcytic anemia between *H. pylori* case 71.6% (N; 48, 95% CI; 0.07–0.33) and *H. pylori* control group 46.1% (N; 18, 95% CI; 0.15–0.30) was computed. However, the prevalence of IDA in *H. pylori*-positive individuals 53.8% (N; 21, 95% CI; 0.15–0.30) was not significantly (*p* > 0.05) different from that of the control group in the age <30 years category (Table 3). Similarly, the proportion of microcytic anemia was also not significantly (*p* > 0.05) different from the control group. The magnitude of the prevalence of IDA, anemia, and microcytic anemia was found to be statistically significant in the rest of the demographic and age categories (Table 2). Prevalence of different types of anemia computed based on marginal distribution was 67.7% IDA (Figure 5a), 35% anemia (Figure 5b), 43% microcytic anemia, and 57% normocytic anemia (Figure 5c). The overall prevalence of *H. pylori*-infected study participants in all five hundred and ten participants was recorded in this study to be 51% (Figure 5a–c).

### 3.4. H. pylori Infection Associated Impact on the Status of IDA, Anemia, and Anemia Morphotype in All the Study Participants (n = 510)

We explained the association of *H. pylori* infection with the development of anemia, IDA, in particular, and anemia morphotype by executing logistic regression analyses. The strength of the association of the demographic and vital hematological parameters with *H. pylori* infection was also determined by undertaking regression analyses. The odds of being infected with *H. pylori* decrease by a factor of 0.97 with one unit increase in the concentration of hemoglobin (AOR; 0.969, 95% CI; 0.688–1.340, *p* < 0.05) compared to the control (uninfected) group (Table 4). Additionally, the odds of having an infection of *H. pylori* bacteria are less likely by a factor of 0.993 with an increment of one unit of ferritin (AOR; 0.993, 95% CI; 0.99–0.997, *p* < 0.001) level in comparison to the uninfected individual (Table 4). Additionally, 28.6% higher odds of being infected with *H. pylori* bacteria were recorded with one unit increase in WBC counts compared to the control group (uninfected group) Table 4. The odds of developing IDA in individuals with *H. pylori* infection was 206.1% higher than in participants without infection (AOR; 3.061, 95% CI; 2.135–4.416, *p* < 0.001). Moreover, the odds of having anemia in *H. pylori*-infected was higher by a factor of 4.983 in comparison to the uninfected participants (AOR; 4.98, 95% CI; 3.089–8.308, *p* < 0.001), as mentioned in Table 5. The odds of developing microcytic anemia as compared to normocytic anemia in individuals with infection was 229% more likely against the control (*H. pylori* uninfected) category (AOR; 3.289, 95% CI; 2.213–4.949, *p* < 0.001), which is reported in Table 5. Significantly decreased odds of MCV were found to be associated with *H. pylori* infection compared to uninfected with an increase in MCV value (AOR; 0.958, 95% CI; 0.909–1.006, *p* < 0.05). An insignificant (*p* > 0.05) association of age with *H. pylori* infection was observed (AOR; 0.958, 95% CI; 1.04–1.5, *p* > 0.05). The odds of having *H. pylori* infection against uninfected decreases by 58.4% with one unit increase in RBC count (AOR; 0.416, 95% CI; 0.181–0.953, *p* < 0.05). The strength of association of gender with *H. pylori* infection was strong as the odds of males being infected were 5.5 times more likely than that of female study participants (AOR; 5.503, 95% CI; 3.225–9.647, *p* < 0.001).

## 4. Discussion

Ubiquitous *H. pylori* infection [40] and anemia [41] have been major public health concerns worldwide. Iron deficiency anemia (IDA) is considered one of the most prevalent forms of anemia [42]. Saju et al. contemplated the remarkable IDA burden in the developing world, which affects the human health of millions [43]. Unexplained IDA has been reported in various literature; many investigations suggested *H. pylori* as one of the causative elements of unexplained IDA [43]. Muhsen et al. referred to the association of iron stores and IDA with *H. pylori* infection in their meta-analysis [44]. Similar observations have also been reported in the investigations accomplished by Zhang et al., who demonstrated the improvement in iron deficiency of the *H. pylori*-infected person following complete eradication of the infection [45], and by Wenzhen et al., who showed the association of *H. pylori* infection with an event of IDA [46]. The association of iron deficiency and/or iron deficiency anemia with *H. pylori* infections in both children and adolescents has been reported in various meta-analyses, which show the impact of *H. pylori* infection on the hematological parameters across the age groups [47]. Kato et al. also explained the recurrent and refractory nature of iron deficiency and IDA in *H. pylori*-infected individuals [47]. In addition, *H. pylori*-associated IDA in children has also been reported [48]. In a correlational study on pediatric subjects (n = 542), Lupu et al. inferred a significant association of *H. pylori* infection with the depletion of iron and the development of iron deficiency anemia in children [48], which implies that the need for preventive clinical measures is of paramount importance, especially in a severe case of iron deficiency anemia even in case of children. Kishore et al. also demonstrated a significant association of serum iron levels with the *H. pylori* infection, which infers the impact of *H. pylori* infection on the depletion of iron storage, iron level, and development of refractory to severe IDA [35]. We undertook a retrospective case–control (observational) investigation to gain insightful findings about the association of *H. pylori* infection with iron deficiency anemia in the study participants (n = 510) of the Asri region of Saudi Arabia. Mean ± SD of the three most significant parameters, Hb, MCV, and ferritin, were measured to be 44.0 ± 13.58, 13.84 ± 2.49, and 83.02 ± 8.31, respectively, in this study because the operational definition of anemia, IDA, and morphotype was based on Hb, ferritin, and MCV level. Nasif et al., in a cross-section study, carried out in Makkah, Saudi Arabia, measured different components of CBC, ferritin, and serum iron to establish the impact of *H. pylori* infection on hematological parameters of study participants (n = 79) [49].

We found that the mean ± SD of hemoglobin in the *H. pylori*-infected subject was 13.26 ± 2.92, which was significantly (*p* < 0.001) lower than that in the *H. pylori*-uninfected subject 14.42 ± 1.75 (Table 2), which is corroborated by the finding of Tanous et al. who described the improvement in the Hb level of the patients following eradication of *H. pylori* infection [50]. Fotia et al. also demonstrated the increased Hb level in *H. pylori*-negative study individuals [51], which conforms with our result. A retrospective study undertaken by Lee et al. explained a statistically significant difference (*p* = 0.019) in mean Hb (14.2 ± 1.69 versus 14.59 ± 1.39) of *H. pylori*-infected subject in comparison to the uninfected control group, which corroborates with our result [52]. Our result conforms with various other investigations accomplished by Mawafy et al. in Palestine [53]. Rahman et al. in Egypt [54], and Zuberi et al. in Pakistan [55]; however, our result is not consistent with the findings of a study carried out in Bangladesh, Dhaka, by Rahman et al., who described that there is no statistically significant difference in the level of Hb in *H. pylori*-infected as compared to the control group [54], and the deviation in our finding from their study report could be explained by the variation in the sample size, population under study, and methods to diagnose the *H. pylori* infection. *H. pylori* bacteria sequester and interfere with the iron absorption following the *H. pylori*-associated chronic gastritis [56] may lead to the lowering of Hb concentration in *H. pylori*-infected individuals. Moreover, hemorrhagic gastritis-dependent iron loss [57] and actively bleeding peptic ulceration may also contribute to depression in the Hb level of *H. pylori*-infected patients.

We contemplated the significant difference (*p* < 0.001) in the level of MCV measured as *H. pylori* positive versus negative (81.29 ± 9.13, 95% CI; 2.12–4.93 vs. 84.82 ± 6.93, 95% CI; 2.12–4.93), which is consistent with a study reported on the relationship of hematological parameters such as MCV (86.149 ± 2.19 vs. 87.30 ± 3.139, *p* < 0.0001), with the event of *H. pylori* infection published by Nasif et al. [49]. Additionally, the MCV level in *H. pylori*-positive was significantly lower in comparison to the control described by our study, which conforms to the findings of studies carried out by Saler et al. in Turkey [58] and Kibru et al. in Ethiopia [59]. However, findings contradictory to our result have also been reported by Mwafy et al. in their study accomplished in Palestine [53]. The contradictory result could be explained by different sample sizes and variations in population. Our results also unravel the statistically significant difference in the level of RBC, MCH, and MCHC values among *H. pylori* case and control, which is corroborated by the results of a study carried out in Ethiopia by Haile et al. [33]. Furthermore, our study elucidated the statistically significant difference (*p* < 0.001) in the level of ferritin *H. pylori* case as compared to the *H. pylori* control category (48.11 ± 63.75, 95% CI; 11.30–34.83 vs. 71.17 ± 71.14, 95% CI; 11.30–34.83) (Table 2), which is substantiated by the finding of Lee et al., who demonstrated the ferritin level in *H. pylori* case as 121.7 ± 106.9 vs. 151.8 ± 107.8 (*p* = 0.027) [52]. In addition, Tanous et al. described the lower level of ferritin in *H. pylori*-positive individuals as compared to the control category, which improvised following effective eradication of the *H. pylori* infection [50]. Miernyk et al. described the statistically significant difference in the geometric mean of ferritin level in an individual with *H. pylori* infection as compared to an uninfected group of individuals [60]. A significant association with lower serum ferritin levels in *H. pylori*-infected individuals has been reported that conforms with our results; however, contrary to that, higher significantly higher ferritin levels reported in *H. pylori*-infected individuals by Kishore et al. [35].

As per our findings, the statistically significant depression in the level of Hb and ferritin in the *H. pylori*-positive case category point toward the impact of *H. pylori* infection on anemia and iron deficiency anemia. Positive correlation between MCV and ferritin (r = 0.287, *p* < 0.001), Hb and MCV (r = 0.732, *p* < 0.001), and Hb and ferritin (r = 0.451, *p* < 0.001) in *H. pylori*-infected individuals was deduced to explain the effect of *H. pylori* infection on the development of anemia, iron deficiency anemia, and anemia morphotype (Table 2).

For the overall category, the significant (*p* < 0.001) difference in anemia prevalence (case vs. control) was 78% (N; 90, 95% CI; 0.27–0.44) vs. 21% (N; 24, 95% CI; 0.27–0.44) (Table 3). Haile et al., in their study, reported 92% of anemia prevalence in infected individuals, which is slightly higher than our result [32]. A 65% prevalence of anemia was also reported by Eyoum and Kouitcheu [61], which is close to our findings. Our result was not in conformity with that of Haile et al., who observed anemia proportion in *H. pylori*-positive individuals to be 29.19% [32]. We found the prevalence of anemia incomparable with findings of various other investigations: in Uganda by Asiimwe et al. (19.9%) [62]; Cuba (24.6%) by Pita-Rodríguez et al. [63]; Butajira (26.9%) by Kibru et al. [59]; Karachi (25%) by Abdul et al. [64]; and Brazil (20%) [65]. This variation in the prevalence of anemia could be expounded by differences in sample size, methods adopted, and characteristics of the populations considered for studies. We explicated that the overall prevalence of IDA in case (*H. pylori*-infected) was significantly different by 63.3% (N; 176, 95% CI; 0.18–0.35, *p* < 0.001) from the control group, which was corroborated by the finding of Annibale et al., who described the 61% of IDA prevalence in *H. pylori*-associated gastritis patients [66]. However, the proportion of IDA in *H. pylori*-positive participants was higher than the findings of Eyoum and Kouitcheu [61]. Our finding was also not in conformity with the prevalence (37.5%) of IDA in *H. pylori*-infected participants reported by Rahat and Kamani [67] (38%) and by Monzon et al. [68]. The alteration of iron absorption, gastric physiology, sequestration of iron, depletion of iron stores, and ulcerative gastric bleeding could be major factors for the development of IDA; however, in recent times, several pieces of evidence have suggested that IDA can be developed even in the absence of peptic ulcer eroded gastric bleeding lesion [66], which indicates towards the other possible underlying factors of *H. pylori*-associated IDA. Prevalence of IDA in *H. pylori*-positive individuals (N/%; 21/53.8, 95% CI; 0.15–0.30) was insignificantly (*p* > 0.05) different from that of the control group in the age <30 years category (Table 2), which could be explicated by the difference in the number of participants (age < 30) years. We observed the prevalence of different kinds of anemia computed based on marginal distribution was 67.7 % IDA (Figure 5a), 35% anemia (Figure 5b), 43% microcytic anemia, and 57% normocytic anemia (Figure 5c). Kibru et al. reported all the morphotypes of anemia in the *H. pylori*-infected group: microcytic, normocytic, macrocytic, and macrocytic anemia [59]; however, we observed only normocytic and microcytic anemia morphotypes, which suggests the *H. pylori* infection may cause IDA by decreasing iron absorption and minimizing Hb concentration owing to iron deficiency [62].

Our result showed that *H. pylori* infection (AOR; 4.98, 95% CI; 3.089–8.308, *p* < 0.001) was significantly associated with anemia, which was corroborated by the demonstration of significant association of infection with anemia: (AOR; 1.699, 95% CI; 1.050–2.980, *p* < 0.05), (OR; 1.29, 95% CI; 0.891–1.87, *p* = 0.17) and odds ratio (1.77) by Haile et al. [32], Eyoum and Kouitcheu [61], and Haile et al. [32], respectively. Furthermore, our result is also consistent with various other published reports [69,70,71,72]. Additionally, our result was also in agreement with that of a study carried out in the USA by Cardenas et al. [73]. In addition, a significant association of Hb (AOR; 0.969, 95% CI; 0.688–1.340, *p* < 0.05) and RBC (AOR; 0.416, 95% CI; 0.181–0.953, *p* < 0.05) (Table 4) with *H. pylori* infection measured in our study supports the existence of a positive association of anemia with infection. In this study, we also assessed the significant and strong association of IDA with *H. pylori* infection (AOR; 3.061, 95% CI; 2.135–4.416, *p* < 0.001), which corroborates with the similar findings of Qu et al. [72], who described the enhanced risk of IDA in the infected group (OR; 2.599; 95% CI; 1.50–4.60). Low ferritin level (AOR; 0.993, 95% CI; 0.99–0.997, *p* < 0.001) in the *H. pylori*-infected group was determined (Table 4). Moreover, our result was also in agreement with the significant association of infection with IDA (OR; 1.564, 95% CI; 1.020–2.395, *p* = 0.04) reported by Eyoum and Kouitcheu [61]. Moreover, the relationship between low ferritin and IDA with *H. pylori* infection has also been authored in many studies [43,73,74]; however, some studies did not find an association between infection with low ferritin levels [71,75]. MCV (AOR; 0.958, 95% CI; 0.909–1.006, *p* < 0.05) showed its association with infection in our study, which is corroborated by the observation (*p* = 0.046) of El Demerdash [27]. We observed the occurrence of microcytic anemia to be 229% (AOR; 3.289, 95% CI; 2.213–4.949, *p* < 0.001) more in infected individuals, which is substantiated by another study carried out by Eyoum and Kouitcheu [61]. Primary data on the diagnosis of *H. pylori* infection in the study subjects were obtained based immune-chromatographic stool antigen test. The stool antigen test diagnostic data was not further confirmed by the endoscopy, hematoxylin and eosin/Giemsa staining of biopsies, or rapid urease test (RUT), which highlights the limitations of the present study. Stratification of case and control subjects based on serodiagnosis, histopathological examination, and endoscopy along with stool antigen test is recommended to avoid the misclassification between cases and controls due to occasional disappearance of *H. pylori* from gastric mucosa with severe atrophy, especially in elder subjects.

## 5. Conclusions

Hematological disorders are significant extra-gastric impacts of *H. pylori* infection. Iron deficiency and/or IDA is one of the most important hematological extra-gastric implications of *H. pylori* infection in both children and adolescents. Gastric bleeding, impaired iron absorption, and competition for iron between *H. pylori* and the host are significant factors for iron deficiency and IDA. Although an association of *H. pylori* infection with an imbalance in hematological parameters of the infected individuals has been reported in various literature, the role of the associated virulence factors of the bacterium and the genetic factors of the host in the depletion of iron and the development of IDA in infected individuals remains to be studied. Moreover, detailed studies on *H. pylori* infection-associated hematological disorders in different populations are noteworthy. In conclusion, our study has unraveled the significant degree of association of anemia, iron deficiency anemia (IDA), decreased ferritin level, reduced hemoglobin concentration, and declined MCV values in *H. pylori*-infected as compared to a control group (uninfected group) in the population of Asir region of the kingdom of Saudi Arabia. Although only a few studies have been carried out to establish the association of *H. pylori* infection with the development of different types of anemia in KSA, our finding corroborates with the findings of various already published literature. In addition to the comparatively aggrandized degree of prevalence of anemia, IDA has been determined in the *H. pylori*-infected category. The occurrence of microcytic anemia (an anemia morphotype) was observed to be linked with the infected group as compared to normocytic anemia. Routine monitoring of hematological parameters of the *H. pylori*-infected individuals, along with preventive measures, must be exercised, especially in the case of *H. pylori* patients with chronic gastrointestinal complications, to avoid hematological sequelae in the patients. Additionally, the eradication of *H. pylori* infection could lead to the effective management of infected patients with hematological complications. Furthermore, the impact of *H. pylori*-associated iron deficiency/IDA and growth impairment in the case of children, particularly in underdeveloped countries, needs to be determined. Community-based longitudinal and cohort studies with greater sample sizes could effectuate full insight into *H. pylori*-associated hematological imbalance. A test-and-treat clinical strategy could be applied mainly in the case of asymptomatic individuals to avoid the hematological complications of *H. pylori* infection. The role of various factors, such as bacterial adhesins and invasions, bacterial colonization, cytotoxins, IDA-specific virulence factors, and host factors in the development of IDA, is recommended to be investigated.

## Figures and Tables

**Table 1:** Tabular representation of baseline characteristics of hematological parameters all the participants (n = 510) stratified in six categories.

Variables	Statistics	Categories					
		Overall	Male	Female	Age < 30 (In Year)	Age = 30–60 (In Year)	Age > 60 (In Year)
Age (In year)	MEDIAN (IQR)	43 (53–35.00)	42. (52–33.0)	45 (54–36)	24 (27–19)	44 (51–37)	66 (70–63)
	Mean ± SD	44.0 ± 13.58	43.31 ± 13.81	44.69 ± 13.33	23.01 ± 5.45	44.01 ± 8.10	67.29 ± 5.83
Hb (g/dL)	MEDIAN (IQR)	14.2 (15.6–12.3)	15.6 (16.63–14.4)	13.2 (14.2–11.8)	14.40 (15.25–13.00)	14.3 (15.9–12.3)	13.6 (14.80–12.25)
	Mean ± SD	13.84 ± 2.49	15.60 ± 2.34	12.68 ± 2.05	13.64 ± 2.45	13.94 ± 2.53	13.45 ± 2.21
MCV (fL)	MEDIAN (IQR)	84.02 (89.1–78.1)	86.1 (89.28–79.43)	83.1 (88.6–75.8)	84.5 (87.65–77.3)	84.3 (88.7–78.1)	87.5 (91.05–78.4)
	Mean ± SD	83.02 ± 8.31	84.06 ± 7.76	82.01 ± 8.71	82.44 ± 8.46	82.86 ± 8.14	84.63 ± 9.04
MCH (pg)	MEDIAN (IQR)	27.5 (29.1–25.5)	28.20 (29.20–26.20)	26.80 (28.80–23.93)	26.8 (29.25–26)	27.5 (29–25.5)	28.3 (29.8–24.8)
	Mean ± SD	26.8 ± 3.36	27.33 ± 3.09	26.29 ± 3.53	26.63 ± 3.26	26.73 ± 3.22	27.39 ± 4.15
MCHC (g/dL)	MEDIAN(IQR)	31.9 (32.9–30.9)	32.30 (33.20–31.40)	31.50 (32.60–30.60)	31.95 (32.98–30.93)	31.90 (32.90–30.90)	31.8 (32.9–30.35)
	Mean ± SD	31.75 ± 2.1	32.09 ± 1.84	31.42 ± 2.28	31.73 ± 2.05	31.74 ± 1.90	31.82 ± 3.1
Platelets (10^9^/L)	MEDIAN (IQR)	262.0 (307–220)	242 (284.2–208.0)	279 (335.75–242.25)	272 (306.0–231.0)	263 (314–224)	242 (288.50–207.50)
	Mean ± SD	270.2 ± 74.23	248.9 ± 64.50	291.01 ± 77.28	272.0 ± 62.24	273.17 ± 77.03	249.90 ± 66.80
WBC (10^9^/L)	MEDIAN (IQR)	5.69 (6.9–4.74)	5.53 (6.54–4.66)	5.89(7.09–4.84)	5.37 (6.55–4.67)	5.77(6.96–4.79)	5.57(6.60–4.67)
	Mean ± SD	5.99 ± 1.71	5.88 ± 1.69	6.10 ± 1.72	5.83 ± 1.76	6.07 ± 1.75	5.68 ± 1.36
RBC (10^12^/L)	MEDIAN (IQR)	5.19 (5.66–4.68)	5.58 (5.88–5.18)	4.87 (5.21–4.48)	5.14 (5.52–4.72)	5.25 (5.70–4.71)	5 (5.41–4.39)
	Mean ± SD	5.14 ± 0.75	5.47 ± 0.73	4.83 ± 0.61	5.09 ± 0.77	5.19 ± 0.73	4.90 ± 0.75
Ferritin (Microgram/L)	MEDIAN (IQR)	24.5 (89–12)	78.65 (143–22.23)	14.85 (31.75–7.8)	24.8 (90.15–11.00)	23.70 (92.00–12.00)	28.20 (85.00–12.75)
	Mean ± SD	59.42 ± 68.37	92.95 ± 78.66	26.67 ± 32.21	63.43 ± 77.54	59.76 ± 68.62	52.92 ± 55.40

**Table 2:** Comparative status of hematological parameters by *Helicobacter pylori* infection status (Welch two sample *t*-test and SD function).

Overall Participants					
Haematological Parameter	*Helicobacter pylori* Infection Status				*p*-Value
	*H. pylori* Negative		*H. pylori* Positive		
	Mean ± SD	95% CI	Mean ± SD	95% CI	
Haemoglobin (g/dL)	14.42 ± 1.75	(0.74–1.57)	13.26 ± 2.92	(0.74–1.57)	*p* < 0.001
Ferritin (µg/L)	71.17 ± 71.14	(11.30–34.83)	48.11 ± 63.75	(11.30–34.83)	*p* < 0.001
MCV (fL)	84.82 ± 6.93	(2.12–4.93)	81.29 ± 9.13	(2.12–4.93)	*p* < 0.001
MCH (pg)	27.36 ± 2.99	(0.54–1.69)	26.25 ± 3.59	(0.54–1.69)	*p* < 0.001
MCHC (g/dL)	32.25 ± 1.91	(0.63–1.34)	31.26 ± 2.16	(0.63–1.34)	*p* < 0.001
RBC (10^12^/L)	5.29 ± 0.58	(0.16–0.41)	4.99 ± 0.85	(0.16–0.41)	*p* < 0.001
Male participants					
Haemoglobin (g/dL)	15.59 ± 1.51	(0.47–1054)	14.58 ± 2.73	(0.47–1054)	*p* < 0.001
Ferritin (µg/L)	124.88 ± 73.03	(37.86–74.69)	68.60 ± 74.15	(37.86–74.69)	*p* < 0.001
MCV (fL)	85.26 ± 6.64	(0.24–3.98)	83.14 ± 8.42	(0.24–3.98)	*p* < 0.05
MCH (pg)	27.64 ± 2.76	(0.18–1.32)	27.08 ± 3.30	(0.18–1.32)	*p* > 0.05
MCHC (g/dL)	32.44 ± 1.57	(0.17–1.05)	31.82 ± 1.99	(0.17–1.05)	*p* < 0.05
RBC (10^12^/L)	5.65 ± 0.49	(0.17–0.50)	5.31 ± 0.84	(0.17–0.50)	*p* < 0.001
Female participants					
Haemoglobin (g/dL)	13.52 ± 1.33	(1.39–2.34)	11.65 ± 2.28	(1.39–2.34)	*p* < 0.001
Ferritin (µg/L)	29.65 ± 30.0	(1.40–14.60)	23.06 ± 34.3	(1.40–14.60)	*p* > 0.05
MCV (fL)	84.48 ± 7.15	(3.36–7.55)	79.02 ± 9.47	(3.36–7.55)	*p* < 0.001
MCH (pg)	27.15 ± 3.15	(1.06–2.76)	25.23 ± 3.67	(1.06–2.76)	*p* < 0.001
MCHC (g/dL)	32.10 ± 2.13	(0.99–2.05)	30.58 ± 2.16	(0.99–2.05)	*p* < 0.001
RBC (10^12^/L)	5.00 ± 0.47	(0.24–0.54)	4.60 ± 0.69	(0.24–0.54)	*p* < 0.001
Age category (Age < 30)					
Haemoglobin (g/dL)	14.34 ± 1.09	(0.33–2.90)	12.71 ± 3.32	(0.33–2.90)	*p* < 0.05
Ferritin (µg/L)	67.31 ± 84.82	(27.33–45.45)	58.25 ± 67.68	(27.33–45.45)	*p* > 0.05
MCV (fL)	86.05 ± 6.00	(4.64–12.22)	77.62 ± 8.92	(4.64–12.22)	*p* < 0.001
MCH (pg)	27.75 ± 2.33	(1.06–4.17)	25.13 ± 3.72	(1.06–4.17)	*p* < 0.01
MCHC (g/dL)	32.24 ± 1.22	(0.12–2.25)	31.05 ± 2.68	(0.12–2.25)	*p* < 0.05
RBC (10^12^/L)	5.19 ± 0.54	(0.16–0.65)	4.94 ± 0.99	(0.16–0.65)	*p* > 0.05
Age category (Age = 30–60)					
Haemoglobin (g/dL)	14.52 ± 1.86	(0.64–1.62)	13.39 ± 2.92	(0.64–1.62)	*p* < 0.001
Ferritin (µg/L)	73.34 ± 70.69	(12.25–39.72)	47.35 ± 64.38	(12.25–39.72)	*p* < 0.001
MCV (fL)	84.27 ± 6.88	(1.08–4.30)	26.34 ± 8.96	(1.08–4.30)	*p* < 0.01
MCH (pg)	27.15 ± 2.75	(0.17–1.46)	26.34 ± 3.55	(0.17–1.46)	*p* < 0.05
MCHC (g/dL)	32.23 ± 1.48	(0.56–1.30)	31.29 ± 2.11	(0.56–1.30)	*p* < 0.001
RBC (10^12^/L)	5035 ± 0.56	(0.17–1.45)	5.04 ± 0.83	(0.17–1.45)	*p* < 0.001
Age category (Age > 60)					
Haemoglobin (g/dL)	13.93 ± 1.65	(0.15–2.00)	13.00 ± 2.56	(0.15–2.00)	*p* > 0.05
Ferritin (µg/L)	63.36 ± 53.12	(7.68–47.55)	43.43 ± 56.50	(7.68–47.55)	*p* > 0.05
MCV (fL)	86.49 ± 8.01	(0.90–8.02)	82.93 ± 9.69	(0.90–8.02)	*p* > 0.05
MCH (pg)	28.12 ± 4.64	(0.72–3.49)	26.73 ± 3.57	(0.72–3.49)	*p* > 0.05
MCHC (g/dL)	32.41 ± 3.97	(0.47–2.74)	31.27 ± 1.91	(0.47–2.74)	*p* > 0.05
RBC (10^12^/L)	5.02 ± 0.61	(0.13–0.60)	4.78 ± 0.84	(0.13–0.60)	*p* > 0.05

**Table 3:** Magnitude of anemia, IDA, and morphotype by *H. pylori* infection status in Asir region of Saudi Arabia (2-sample test for equality of proportions and Pearson’s Chi-squared test to assess *p*-value).

Overall Participants					
Anemia Status	*Helicobacter pylori* Infection Status				*p*-Value
	*H. pylori* Negative		*H. pylori* Positive		
	N (%)	95% CI	N (%)	95% CI	
Anemic	24 (21%)	(0.27–0.44)	90 (78%)	(0.27–0.44)	*p* < 0.001
Iron deficiency anemia	102 (36.6%)	(0.18–0.35)	176 (63.3%)	(0.18–0.35)	*p* < 0.001
Anemia morphotypes (Microcytic)	47 (29%)	(0.19–0.37)	112 (70.4%)	(0.19–0.37)	*p* < 0.001
Male participants					
Anemic	5 (12%)	(0.25 0.49)	36 (87%)	(0.25 0.49)	*p* < 0.001
Iron deficiency anemia	7 (7%)	(0.44–0.63)	81 (92%)	(0.44–0.63)	*p* < 0.001
Anemia morphotypes (Microcytic)	19 (28%)	(0.07–0.33)	48 (71.6%)	(0.07–0.33)	*p* < 0.01
Female participants					
Anemic	19 (26%)	(0.27–0.52)	54 (73.9%)	(0.27–0.52)	*p* < 0.001
Iron deficiency anemia	95 (50%)	(0.04–0.30)	95 (50%)	(0.04–0.30)	*p* < 0.05
Anemia morphotypes (Microcytic)	28 (30.4%)	(0.25–0.49)	64 (69.5%)	(0.25–0.49)	*p* < 0.001
Female Age category (Age < 30)					
Anemic	2 (28.5%)	(0.31–0.75)	12 (85.7%)	(0.31–0.75)	*p* < 0.001
Iron deficiency anemia	21 (53.8%)	(0.15–0.30)	18 (46.1%)	(0.15–0.30)	*p* > 0.05
Anemia morphotypes (Microcytic)	4 (18.1%)	(0.36–0.77)	18 (81.8%)	(0.36–0.77)	*p* < 0.01
Female Age category (Age = 30–60)					
Anemic	18 (21.9%)	(0.22– 0.43)	64 (78%)	(0.22–0.43)	*p* < 0.001
Iron deficiency anemia	70 (65.6%)	(0.15 – 0.30)	134 (34.3%)	(0.15 – 0.30)	*p* < 0.001
Anemia morphotypes (Microcytic)	38 (31.9%)	(0.20–0.38)	81 (15.1%)	(0.20–0.38)	*p* < 0.001
Female Age category (Age > 60)					
Anemic	4 (22.2%)	(0.11–0.59)	14 (77.7%)	(0.11–0.59)	*p* < 0.05
Iron deficiency anemia	11 (31.4%)	(0.13–0.59)	24 (68.5%)	(0.13–0.59)	*p* < 0.01
Anemia morphotypes (Microcytic)	5 (22.2%)	(0.11–0.59)	13 (77.7%)	(0.11–0.59)	*p* > 0.05

**Table 4:** Results of univariate and bivariate regression analyses (logistic model) to assess *H. pylori*-associated anemia.

Demographic and Haematological Parameters by *H. pylori* Infection Status	Univariate BLR			Bivariate BLR		
	Unadjusted/COR			Adjusted AOR		
	COR	95% CI	*p*-Value	AOR	95% CI	*p*-Value
Age	1.211	1.03–1.4	*p* > 0.051	1.255	1.04–1.5	*p* > 0.051
Gender	5.402	3.132–9.546	*p* < 0.001	5.503	3.225–9.647	*p* < 0.001
Haemoglobin	0.819	0.758–0.883	*p* < 0.001	0.969	0.688–1.340	*p* < 0.05
Ferritin	0.995	0.992–0.997	*p* < 0.001	0.993	0.990–0.997	*p* < 0.001
MCV	0.948	0.926–0.969	*p* < 0.001	0.958	0.909–1.006	*p* < 0.05
MCH	0.901	0.852–0.951	*p* < 0.001	0.959	0.821–1.131	*p* > 0.05
MCHC	0.764	0.688–0.844	*p* < 0.001	0.768	0.694–1.021	*p* > 0.05
RBC	0.579	0.450–0.738	*p* < 0.001	0.416	0.181–0.953	*p* < 0.05
PLT	0.999	0.996–1.001	*p* > 0.05	0.996	0.993–0.999	*p* > 0.05
WBC	1.123	1.013–1.249	*p* > 0.051	1.286	1.138–1.460	*p* < 0.001

Reference category: female; COR = crude odds ratio; AOR = adjusted odds ratio.

**Table 5:** Results of univariate and bivariate regression analyses (logistic model) to assess the association of *H. pylori* infection with anemia.

Anemia Status *by H. pylori* Infection	Univariate BLR			Bivariate BLR		
	Unadjusted/COR			Adjusted OR		
	COR	95% CI	*p*-Value	AOR	95% CI	*p*-Value
Anemia	4.985	3.093–8.308	*p* < 0.001	4.983	3.089–8.308	*p* < 0.001
IDA	3.040	2.122–4.381	*p* < 0.001	3.061	2.135–4.416	*p* < 0.001
Anemia morphotypes (Microcytic)	3.269	2.200–4.914	*p* < 0.001	3.289	2.213–4.949	*p* < 0.001

Reference category: non-anemic; uninfected; non-iron deficiency; and normocytic anemia. COR = crude odds ratio; AOR = adjusted odds ratio.

## Data Availability

The data supporting this study’s findings are not publicly available to protect privacy of the research participant but are available from corresponding author.
